# Multi-omics approach to personalised treatment: insights into thrombus-derived exosome regulation in cardiomyocyte ferritinophagy

**DOI:** 10.3389/fimmu.2025.1607355

**Published:** 2025-08-28

**Authors:** Youfu He, Liqiong Ai, Yu Zhou, Jing Huang, Xiangshu Long, Qiang Wu

**Affiliations:** ^1^ Medical College, Guizhou University, Guiyang, Guizhou, China; ^2^ Department of Cardiology, Guizhou Provincial People’s Hospital, Guiyang, Guizhou, China; ^3^ Department of Cardiology, Affiliated People’s Hospital of Guizhou University, Guizhou, China; ^4^ Department of Dentistry, Guiyang Borui Dental Clinic, Guiyang, Guizhou, China

**Keywords:** thrombus-derived exosome, M6A, LncRNA FENDRR, ferritinophagy, p53, NCOA4, single cell analysis, macrophages

## Abstract

**Background:**

Type 1 myocardial infarction (T1MI) is an acute ischemic event triggered by the rupture of a coronary atherosclerotic plaque. The pathogenesis of T1MI is highly complex, involving disturbances in iron metabolism, cell apoptosis, immune activation, and inflammatory responses. In recent years, ferritinophagy, a novel autophagic mechanism regulating iron homeostasis, has attracted increasing attention for its role in cardiovascular diseases. However, its precise involvement in T1MI remains to be fully elucidated. This study aims to systematically analyse the mechanism of ferritinophagy in T1MI and explore its potential connection to immune and inflammatory responses.

**Methods:**

Exosomes were isolated from coronary thrombi of T1MI patients and subjected to comprehensive transcriptomic profiling. Differentially expressed lncRNAs and mRNAs were validated through functional assays, including RIP, FISH, ChIP, and m6A methylation experiments. Cardiomyocyte models and integrated bulk and single-cell RNA sequencing were used to clarify cellular context and regulatory networks, with particular emphasis on YTHDF family proteins. Bioinformatics analyses, including GO and KEGG, were employed for pathway annotation.

**Results:**

Electron microscopy confirmed the presence of exosomes in coronary thrombi. Thrombus-derived exosomes (TEs) induced pronounced ferritinophagy in cardiomyocytes, evidenced by increased autophagosomes, ROS, apoptosis, and iron overload, with these effects ameliorated by the ferroptosis inhibitor Fer-1. Transcriptomic and functional analyses identified lncRNA FENDRR as highly enriched in TEs, with FENDRR and P53 acting in concert to regulate NCOA4 and system Xc–. Mechanistically, FENDRR directly binds P53, and both upregulate m6A modification in cardiomyocytes, specifically through upregulation of YTHDF1 and downregulation of YTHDF3. Inhibition of either FENDRR or P53 reverses these changes. Single-cell RNA-seq analysis revealed significant upregulation of TP53, NCOA4, and YTHDF1, alongside downregulation of YTHDF3 in macrophages from plaque tissue, linking ferritinophagy, autophagy, and immune-inflammatory responses.

**Conclusion:**

This study is the first to reveal the critical role of the “FENDRR–m6A–NCOA4” regulatory axis as a critical mediator of ferritinophagy in T1MI. It also suggests that immune cells may participate in the immune-inflammatory response associated with myocardial injury via ferritinophagy. Our research provides multi-omics evidence of the interaction between iron homeostasis, immunity, and inflammation in T1MI, offering potential therapeutic strategies for targeting ferritinophagy and related RNA modification pathways.

## 1 Introduction

Type 1 myocardial infarction (T1MI), which is precipitated by coronary thrombosis, remains a major cause of morbidity and mortality worldwide, and represents a significant clinical challenge (1). Acute coronary thrombosis initiates a cascade of local inflammation, hypoxia, and immune cell infiltration, leading to a spectrum of severe complications, including T1MI itself, cardiogenic shock, cardiac rupture, and sudden cardiac death. Thus, elucidating the mechanisms underlying coronary thrombosis formation and its downstream effects on cardiac pathology is of critical importance. Despite advances in medical science, the biology of thrombi—the central trigger of acute myocardial infarction (AMI)—remains poorly understood. In particular, how thrombi and the substances they produce or transport, such as exosomes, modulate cardiomyocyte pathophysiology after AMI is still largely unexplored. Therefore, a deeper understanding of the interplay between coronary thrombosis, T1MI, and the subsequent progression of cardiac disease is urgently needed.

Exosomes, as critical mediators of intercellular communication, have been extensively studied and are widely recognised for their functional significance. These vesicles, which possess a phospholipid bilayer and range from approximately 30–150 nm in diameter, are secreted by living cells and serve as carriers for diverse molecular cargo, enabling them to modulate the behaviour of nearby tissues or even distant organs (2). Numerous studies have demonstrated that exosomes derived from macrophages (3), platelets (4) and cardiac progenitor cells (5), can influence cardiomyocyte function within infarcted myocardium following AMI. Notably, inflammation and immune response appear to be central to these processes. For example, Leroyer et al. (6) reported the presence of vesicular structures in cells and tissues adjacent to coronary arteries. However, it remains unclear whether thrombi responsible for T1MI harbour exosomes and, if so, how these exosomal components affect cardiomyocyte pathophysiology after AMI. Addressing this question is of urgent scientific interest.

The cascade amplification properties of non-coding RNAs(ncRNAs) have increasingly drawn attention to their roles in cardiovascular disease, particularly in the context of exosome-mediated molecular signaling. In recent years, multiple studies have linked cardiovascular pathology, exosomes, and ncRNAs, with some suggesting that certain long ncRNAs (lncRNAs) could serve as novel biomarkers for cardiovascular conditions (7). Among these, lncRNAs, such as Fetal lethal non-coding developmental regulatory RNA (FENDRR) (8), ZNFX1 antisense RNA 1 (ZFAS1) (9), and nuclear paraspeckle assembly transcript 1 (NEAT1) (10) have been found to participate in a variety of pathophysiological processes, including AMI and heart failure (HF). Moreover, several studies have highlighted the exosome-associated lncRNAs to modulate the development of cardiac diseases. In addition, exosomes from various cell types have been implicated in the regulation of m6A methylation in cells and tissues via lncRNAs (11), influencing both cellular damage and repair mechanisms following AMI (12). N6-methyladenosine (m6A) modification is the most prevalent and functionally significant mRNA modification in mammals, accounting for accounting for nearly half of all methylated ribonucleotides (13). A growing body of evidence supports a critical role for m6A modifications in cardiovascular disease, particularly in pathways associated with apoptosis, autophagy, and ferritinophagy (14).

Ferritinophagy is a form of regulated cell death characterized by iron-dependent accumulation of cellular reactive oxygen species (ROS), which occurs when glutathione (GSH)-dependent lipid peroxidation repair systems are impaired (15). The nuclear receptor coactivator 4 (NCOA4) has emerged as a principal effector in ferritinophagy and is now widely acknowledged in the cardiovascular field as a critical regulator of this pathway (16, 17). Recent research indicates that ferritinophagy plays an essential role in metabolic regulation in cancer cells (18) and is intimately linked to inflammatory responses in cardiovascular disease (19). This is especially pertinent in acute settings such as T1MI, where immune cell infiltration—most notably by macrophages—is pronounced within injured myocardial tissue. Through the reprogramming of iron metabolism, these immune cells can amplify inflammatory signalling via ferritinophagy, thereby promoting cytokine release and aggravating tissue injury (20). Thus, ferritinophagy is increasingly viewed as a vital link between disturbances in iron homeostasis and immune-mediated inflammation. The dynamic changes of ferritinophagy within the immune microenvironment deserve further exploration (21).

In this study, we systematically isolated and characterized exosomes derived from coronary thrombi in patients with T1MI. By employing a multi-omics approach—integrating bulk and single-cell transcriptomics with epigenetic profiling—we investigated the potential contribution of these exosomes to myocardial injury. Our analyses centred on ferritinophagy, an autophagy pathway closely related to iron metabolism, and revealed its aberrant activation in T1MI, accompanied by remodeling of ferroptotic and immune-inflammatory responses. Notably, we identified FENDRR as a pivotal lncRNA regulating the expression of NCOA4, a central mediator of ferritinophagy, through m6A modification, thereby establishing the “FENDRR–m6A–NCOA4” regulatory axis. At the immune level, single-cell RNA sequencing revealed substantial upregulation of ferritinophagy-related genes—including Tumor Protein 53(TP53), NCOA4, and YTH N6-methyladenosine RNA binding protein (YTHDF) 1—in macrophages, indicating a possible synergistic activation of iron metabolism and inflammatory pathways. Together, these findings offer novel mechanistic insights into the interplay between iron homeostasis, immune responses, and myocardial injury in the context of T1MI.

## 2 Methods

### 2.1 Thrombus tissue and serum collection and preparation

Coronary thrombosis, intracoronary thrombi in patients with T1MI, was diagnosed by coronary angiography at the Department of Cardiovascular Medicine of Guizhou Provincial People’s Hospital. Coronary thrombi were removed by catheter aspiration and placed in centrifuge tubes containing 5 mL Phosphate-Buffered Saline(PBS) and stored at 4°C for a maximum of 3 days. Venous thrombosis, thrombi in patients with acute lower extremity thrombosis with onset within 24 h, was diagnosed by angiography at Guizhou Provincial People’s Hospital. Venous thrombi were removed by thromboaspiration and placed in PBS and stored briefly at 4°C, as previously described (22). Rat-derived arterial thrombi were generated using 5% Iron(III) chloride (FeCl_3_) patches on the aorta. Thrombi were then flushed out of the vessel with PBS using a syringe and were stored in PBS at 4°C.

About 5 mL whole blood from the patients with AMI were placed in a pro-coagulation tube, and the serum was extracted by centrifugation at 3000 g for 15 minutes. The serum was stored at -20°C for a maximum of 6 months (23).

### 2.2 Exosome enrichment

We followed the method described by Vella et al. (24) for exosome extraction from the thrombi. Type II collagenase (50 U/mL) and trypsin (20 μg/mL) were added to the coronary thrombi, and 5 mM calcium chloride was added as an enzyme activator, as per the instructions for type II collagenase. The mixture was placed on a constant temperature shaker at 37°C for 1 h, which resulted in complete thrombus dissolution. The mixture was then centrifuged at 300 g for 5 minutes at 4°C to remove incompletely dissolved thrombi and adherent endothelial tissue. Dead cells and cell debris were then removed by centrifugation at 2,000 g for 10 minutes at 4°C. The exosomes were subsequently precipitated by centrifugation at 100,000 g for 70 minutes at 4°C.

To extract exosomes from serum, the serum samples were centrifuged at 2000 g for 10 minutes at 4°C to remove dead cells and cell fragments, followed by centrifugation at 100,000 g for 70 minutes at 4°C to precipitate the exosomes (25, 26).

### 2.3 Cell culture and co-culture with exosomes

In this study, we used both AC16 and H9C2 cardiomyocytes of two different species. All exosomes were diluted to the appropriate concentration (128ng/μL)using PBS and co-cultured with cells cultured to 80% confluency. When exosomes were co-cultured with cells, all FBS was replaced with 5% exosome-free FBS (27).

### 2.4 Immunofluorescence assay

Exosomes (100 µg) were stained with 1 μM Dil and incubated at 37°C in the dark for 30 minutes, and then centrifuged at 100,000 g for 70 minutes. After careful aspiration of the supernatant, 1 mL complete medium containing 10% FBS was added to the precipitate, blown and mixed well. The mixture was then added to the cell culture dish and co-cultured with the corresponding cells for 24 h. The cells were then fixed with 4% paraformaldehyde, nonspecific antigens were blocked with goat antiserum, and the cells in each group were incubated with the corresponding marker antibody overnight. Secondary antibodies with FITC fluorescent labels were then added and incubated in the dark for 1 h. After DAPI staining, fluorescence was detected using a fluorescence microscope(BX60, Olympus, Japan) (28).

### 2.5 Rat T1MI model

Rats (n = 3) were fixed on the operating table, and the chest was opened. A paper sheet infiltrated with 5% FeCl_3_ was attached to the left anterior descending (LAD) artery (29). Significant ST-segment elevation could be seen on the electrocardiogram after approximately 10–20 seconds, indicating that the T1MI model had been successfully established.

### 2.6 Establishment of an acute hypoxia cardiomyocyte model

Cardiomyocytes were cultured to 80% confluency. The culture medium was replaced, and liquid paraffin was dripped on top of the medium until the culture dish was completely covered (30). The cells were then placed in a carbon dioxide incubator for 2 h (n = 3).

### 2.7 Construction of Small Interfering RNA (siRNA) and Short Hairpin RNA for FENDRR and P53

Desalted stealth RNA interference (RNAi) duplexes were designed using Block-iT RNAi Designer (ThermoFisher Scientific, Shanghai, China). The RNAi duplexes against FENDRR (GenBank accession number 033925) had the following sequences:

Sense sequence: GGCUGAUGGUAGAGGUUAAAC

Antisense sequence: UUAACCUCUACCAUCAGCCGG

The RNAi duplexes against TP53 (GenBank accession number XM_008767773.3) had the following sequences:

Sense sequence: GGAUGUUGCAGAGUUGUUAGA

Antisense sequence: UAACAACUCUGCAACAUCCUG

The siRNAs were constructed by Hanheng Biologicals, China.

Short hairpin RNA (shRNA) targeting the lncRNA FENDRR (NR_126575.3, the longer transcript of FENDRR) was designed using the web-based siRNA Wizard software (www.sirnawizard.com). The shRNA sequence was as follows:

Top strand: 5′-CACCGGAGGAAGAGAAGTATCAATTCGAAAATTGATACTTCTCTTCCTCC-3′

Bottom strand: 5′-AAAAGGAGGAAGAGAAGTATCAATTTTCGAATTGATACTTCTCTTCCTCC-3′

### 2.8 RNA-interacting protein immunoprecipitation (RIP) and RNA-protein pulldown

RIP was performed using EZ-Magna RIP RNA-Binding Protein Immunoprecipitation Kit(Millipore, Cat# 17-701) according to the manufacturer’s instructions. RNA was reverse transcribed to cDNA using Hifair^®^ II 1st Strand cDNA Synthesis Kit(Yeasen, Cat# 11119ES60). Taq Pro Universal SYBR qPCR Master Mix(Vazyme, Cat# Q712-02) was used for real-time PCR quantification of the target genes on the 7500 Fast Real-Time PCR System. The FENDRR plasmid was constructed into a pCDNA3.1 vector(Invitrogen, Cat# V79020). RNA for *in vitro* experiments was transcribed using T7 RNA Polymerase Kit(Takara, Cat# 2540A) according to the manufacturer’s instructions. The transcripts were labelled using RNA 3’-End Desthiobiotinylation Kit(Thermo Fisher Scientific, Cat# 20163) according to the manufacturer’s instructions. Pierce™ Magnetic RNA-Protein Pull-Down Kit(Thermo Fisher Scientific, Cat# 20164) was used for RNA-protein pulldown according to the manufacturer’s instructions. The protein pulled down by FENDRR was detected by western blotting with an anti-P53 antibody(Proteintech, Cat# 60283-2-Ig) (31).

### 2.9 Fluorescence *in situ* hybridisation

FISH was performed using Ribo Fluorescent *In Situ* Hybridisation Kit(RiboBio, Cat# C10910). Cy3-labelled lncRNA FENDRR probes were obtained from Sangon Biotech, Shanghai, China(200 nM; Cy3-5′-TCTTCCTCCAGTTCACGTGC-3′). Cardiomyocytes were fixed in 4% paraformaldehyde(Biosharp, Cat# BL539A) for 20 minutes at room temperature, washed three times with PBS(Biosharp, Cat# BL302A), and permeabilised with 0.5% Triton X-100(Sigma-Aldrich, Cat# T8787) for 5 minutes. The cells were then blocked with prehybridisation solution (from the FISH kit) for 30 minutes at 37°C. The prehybridisation solution was removed, and the cells were cultured with the probe hybridisation solution overnight in the dark. Finally, the cells were incubated with DAPI(Beyotime, Cat# C1002) for 15 minutes and analysed using a laser confocal microscope (32).

### 2.10 Chromatin immunoprecipitation assay

Cardiomyocytes were crosslinked with 1% formaldehyde(Sigma-Aldrich, Cat# F8775) for 10 minutes at 37°C, and a ChIP assay was performed using EZ-Magna ChIP™ A/G Chromatin Immunoprecipitation Kit(Merck Millipore, Cat# 17-10086) according to the manufacturer’s instructions. Cells were washed with cold PBS(Biosharp, Cat# BL302A) and suspended in 1 mL PBS containing protease inhibitor cocktail(Beyotime, Cat# P0100). Cells were centrifuged at 1200 × r for 5 minutes at 4°C. Cells were lysed with 300 µL SDS lysis buffer and sheared by sonication at 150 Hz with four sets of 10-sec pulses on ice. An equal amount of chromatin (100 µL) was immunoprecipitated at 4°C overnight. The antibodies used in this assay included anti-P53(Proteintech, Cat# 60283-2-Ig) and normal mouse IgG(Abcam, Cat# ab172730). Immunoprecipitated products were collected after incubation with magnetic beads coupled with anti-IgG or anti-P53 (from the ChIP kit). The beads were washed using a magnetic separation rack, and the bound chromatin was eluted in ChIP Elution Buffer with Proteinase K(Beyotime, Cat# ST535).The recovered DNA fragments were analysed using RT-qPCR. The relative level of FENDRR was normalised to the average level of the IgG group (31).

Primer sequences for FENDRR detection:

Forward: 5′-CGCGGGCTTCTCTACTCTTA-3′

Reverse: 5′-CCTTTTACAAGCGCAGGTTC-3.

### 2.11 Identification of differentially expressed lncRNAs and mRNAs

The Limma package of R software(Version 4.3.2) was used to screen differentially expressed mRNA (DEmRNA), DEmiRNA, and DEcircRNA with adjusted *p*-value thresholds set as < 0.05 and | log2FC |> 1.0 (33). Volcano plots and heat maps of the DERNAs were constructed using the R software (23). The lncRNA annotation and prediction were performed using AnnoLnc (accessed in December 2024, http://annolnc.gao-lab.org/) and lncExpDB (accessed in December 2024, https://ngdc.cncb.ac.cn/lncexpdb/). All bioinformatics analyses were performed using R software (version 4.3.2). Differential expression analysis was conducted with the Limma package (version 3.58.1). Gene Ontology (GO) and Kyoto Encyclopedia of Genes and Genomes (KEGG) enrichment were performed using the clusterProfiler package (version 4.10.0), org.Hs.eg.db (version 3.17.0), enrichplot (version 1.22.0), and ggplot2 (version 3.5.1). Differentially expressed lncRNAs and mRNAs were identified using thresholds of adjusted p < 0.05 and |log2FC| > 1.0. For lncRNA function prediction, lncRNAs with the highest expression levels were cross-referenced with ferroptosis-related pathways using AnnoLnc and lncExpDB, with scoring and functional annotation performed according to the default algorithms of the respective platforms.

### 2.12 GO and KEGG enrichment analysis of differentially expressed genes

GO, including biological processes (BP), cellular components (CC), and molecular functions (MF), KEGG enrichment analysis, and visualisation of DEmRNAs were performed using the R-packages ‘clusterProfiler’, ‘org.Hs.eg.db’, ‘enrichplot’, and ‘ggplot2’ with *p*-value thresholds of < 0.05 (23).

### 2.13 m6A dot blot assays

Total cellular RNA was extracted and diluted to 0.5 ng/µL with sealing solution. Two microliters of the sample were then added to the nitrocellulose (NC) membrane in different groups. After drying the samples, the membrane was sealed with sealing solution for 1 h. The membranes were then repeatedly washed with TBST, and the m6A antibody was added at a dilution of 1:1000 and incubated overnight at 4°C. Sheep anti-rabbit HRP secondary antibody was then added at a 1:200000 dilution and incubated for 2 h. After repeated washing, electrogenerated chemiluminescence (ECL) was used for colour development. After complete colour development, the membranes were stained with methylene blue and photographed.

### 2.14 Single-cell RNA sequencing analysis

Publicly available transcriptome datasets were downloaded from the Gene Expression Omnibus (GEO) database (accession: GSE285775) in November 2024. We analysed the publicly available dataset GSE285775 using R and the Seurat package (version 4.3.0) to process and explore single-cell transcriptomic data. Quality control was carried out to remove cells with over 10% mitochondrial gene expression or fewer than 200 detected genes. The data were then normalised using the NormalizeData function, and highly variable genes were selected with FindVariableFeatures. Dimensionality reduction was performed using principal component analysis (PCA), followed by clustering through FindNeighbors and FindClusters. UMAP was used to visualise the distribution of cell populations. Cell types were identified based on known marker genes and published references. The macrophage population was then isolated using the subset function. Differential expression analysis between groups (AIT vs Plaque) was conducted with the FindMarkers function (34). Genes with an adjusted p-value below 0.05 and an absolute log2 fold change greater than 2 were considered differentially expressed. We then performed Gene Ontology (GO) and pathway enrichment analyses on the differentially expressed genes identified in the macrophage population (35).

### 2.15 Statistical analysis

GraphPad 7 were used for statistical analysis and image rendering of the experimental data, respectively. Comparisons between groups were performed using the independent t-test, and comparisons between multiple groups were performed using one-way Analysis of Variance(ANOVA). The least significant difference(LSD)-t test was used when the variances were homogeneous, and the Dunnett’s T3 test was used when the variances were not homogeneous. Multivariate expression profiles were analysed by repeated measures ANOVA, and *post hoc* tests were conducted using the Bonferroni test. Statistical significance was set as *p* < 0.05.

## 3 Results

### 3.1 Coronary thrombi in patients with T1MI contain exosomes

**Figure 1:**
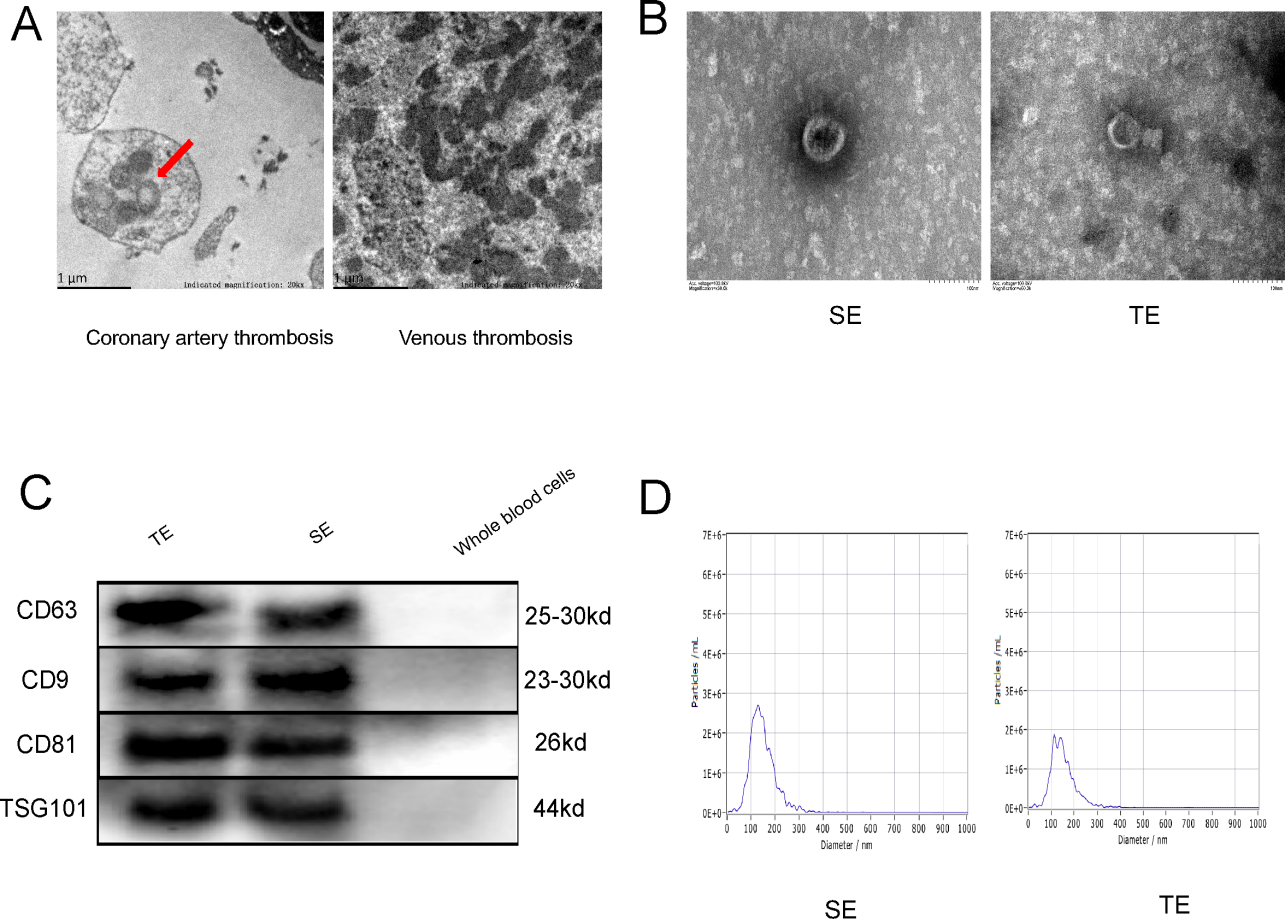
Coronary thrombi in patients with T1MI contains exosomes. **(A)** Electron microscopy (EM) images of a coronary thrombus with an exosome-like structure (red arrow) from a patient with T1MI and a venous thrombus from a patient with acute lower extremity venous thrombosis (approximately 50,000× magnification); **(B)** EM images of exosomes from the serum of patients with AMI (SE) and from coronary thrombi (TE) from the same patients (approximately 150,000× magnification); **(C)** Western blot of exosome markers(n=3); **(D)** Nanoparticle tracking analysis showing the size ranges of the SE from patients with AMI with a peak particle size of 127.6 nm and TEs with a peak particle size of 137.8 nm. SE, serum exosomes from patients with AMI; TE, coronary thrombus-derived exosomes from patients with AMI.

In order to gain insight into the microscopic conditions within the coronary thrombus, the thrombus was subjected to electron microscopy (EM). Exosome-like vesicles were observed in the coronary thrombi of patients with T1MI (**Figure 1A**, red arrows), but not in venous thrombi. This may be attributed to the different causes of thrombosis and the diverse cell types involved. The exosomes isolated from the thrombi and serum samples of patients with T1MI exhibited a consistent biconcave disc shape and measured between 30 and 150 nm in size (**Figure 1B**). The exosomes from both the thrombi and serum samples were positive for surface markers (CD63, CD9, CD81 and TSG101), whereas the whole-blood cells (negative control) did not exhibit significant expression (**Figure 1C**). The greatest exosome sizes detected in the AMI-serum and thrombi were 127.6 and 137.8 nm (**Figure 1D**), respectively, thus falling within the previously defined exosome size range of 30–150 nm. These results confirm the presence of exosomes in coronary thrombi, which were successfully extracted.

### 3.2 Coronary thrombus-derived exosomes induce ferritinophagy in cardiomyocytes

**Figure 2:**
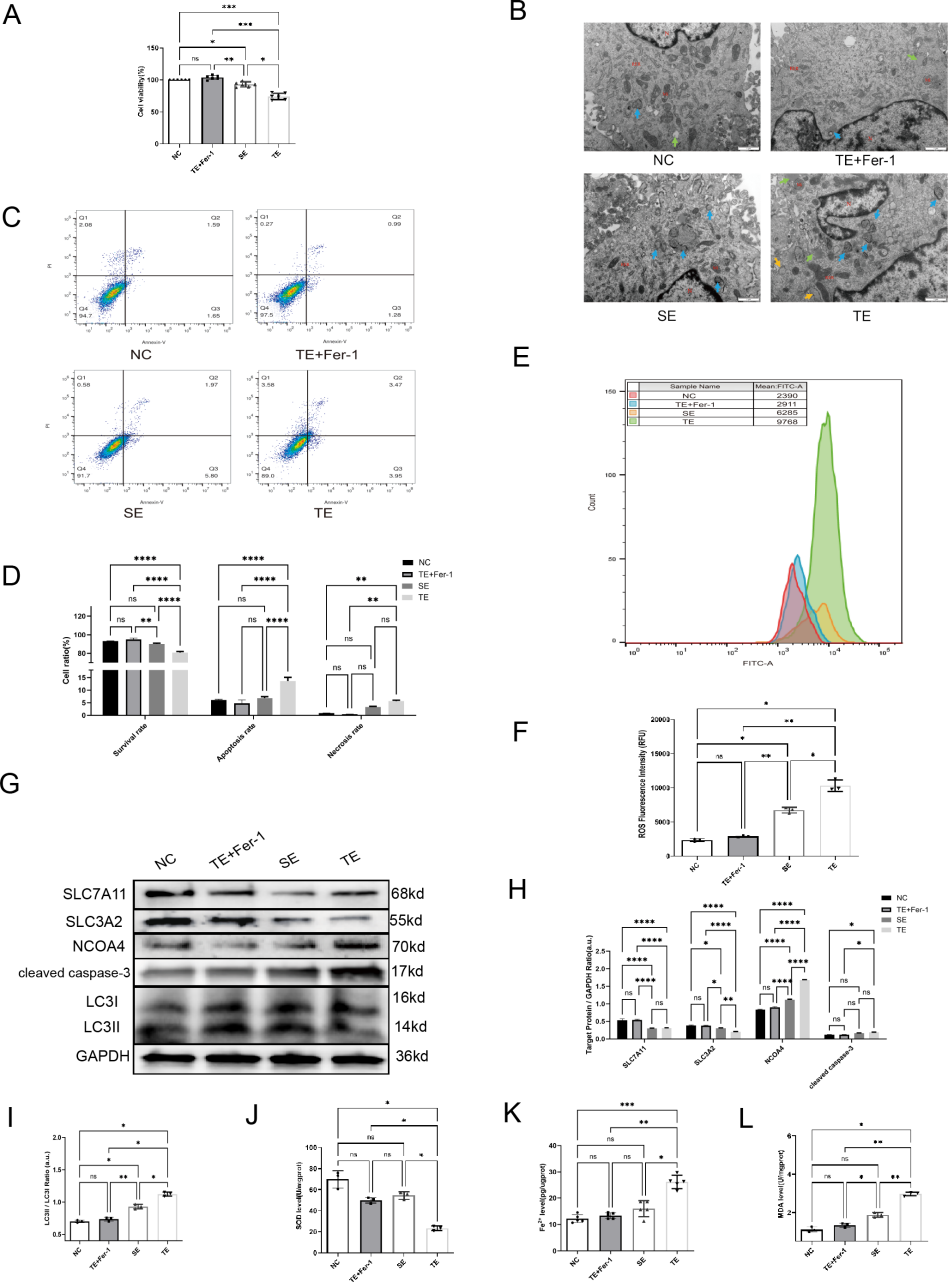
Coronary thrombus-derived exosomes induce ferritinophagy in cardiomyocytes **(A)** Effect of different exosomes on cardiomyocyte viability as measured by CCK-8 assays(n=5); **(B)** EM images of cardiomyocytes after co-culture with exosomes (20,000×) showing microscopic morphology, including the nucleus (N), mitochondria (Mi), rough endoplasmic reticulum (RER), autophagy (blue arrow), and lipid droplets (green arrow); **(C,D)** Flow cytometry results indicating apoptosis of cardiomyocytes after co-culture with exosomes(n=3); **(E, F)** Flow cytometry results indicating intracellular ROS fluorescence intensity in cardiomyocytes after co-culture with exosomes(n=3); **(G-I)** Western blot showing the expression of ferritinophagy-related proteins in cardiomyocytes after co-culture with exosomes(n=3); **(J)** SOD levels in each group(n=5); **(K)** Fe^2+^ levels in each group(n=5); **(L)** MDA levels in each group (n=5). ns: not significant; *p < 0.05; **p < 0.01; ***p < 0.001; ****p < 0.0001.

To investigate exosome endocytosis, we co-cultured Dil-labelled exosomes with AC16 cardiomyocytes. We observed a significant amount of red fluorescence within the cells following co-culture, indicating exosome internalisation by the cells. The results (**Figure 2A**) showed that the viability of cardiomyocytes was significantly decreased in the TE and SE groups compared with the negative control (NC) group (*p*< 0.05). Subsequent EM revealed a significant increase in the number of autophagosomes in cardiomyocytes exposed to SEs and TEs relative to the NC group (**Figure 2B**, blue arrows). Additionally, TE treatment induced apoptosis and necrosis in cardiomyocytes (*p* < 0.05) (**Figures 2C, D**). Furthermore, ROS production was considerably higher in the TE group than in the NC group (*p* < 0.05) (**Figures 2E, F**). Finally, western blot analysis indicated the expression of LC3, cleaved caspase-3, Solute Carrier Family 3 Member 2(SLC3A2), and NCOA4. In the TE group, SLC3A2 and Solute Carrier Family 7 Member 11(SLC7A11) expression were significantly decreased (*p* < 0.05) compared with the NC group, while cleaved caspase-3, NCOA4, and LC3II/I expression were significantly increased (*p* < 0.05) (**Figures 2G–I**). Ferroprostatin-1 (Fer-1), a ferroptosis/ferritinophagy inhibitor, significantly ameliorated most of the aforementioned TE-induced cardiomyocyte injuries (*p* < 0.05). Additionally, we found (**Figures 2J–L**) that TE treatment increased MDA and Fe2^+^ levels (*p* < 0.05), while simultaneously inhibiting SOD levels (*p* < 0.05) in cardiomyocytes. These effects were significantly attenuated by Fer-1 treatment (*p* < 0.05). In light of these findings, it can be posited that TE treatment induces ferritinophagy in cardiomyocytes, a process that is ameliorated by Fer-1.

### 3.3 Bioinformatics analysis of lncRNAs enriched in TEs

Building on the bioinformatics analyses in section 3.3, which identified lncRNA FENDRR as a highly expressed lncRNA in TEs potentially involved in ferroptosis through interaction with P53, we next sought to experimentally validate these findings. To investigate the functional roles of FENDRR and P53 in TE-mediated effects on cardiomyocytes, we conducted *in vitro* studies to assess their expression and regulatory impacts.

**Figure 3:**
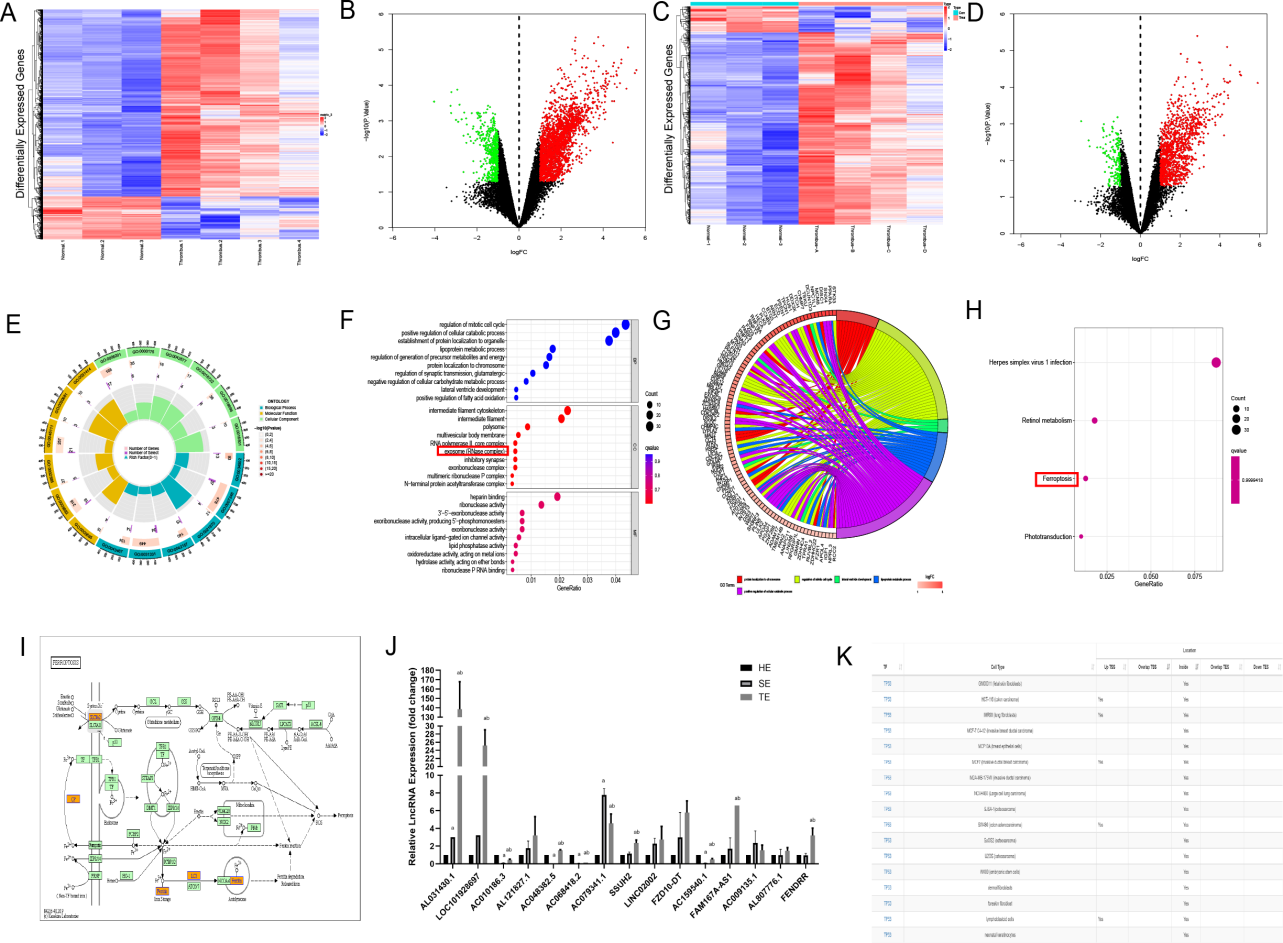
Bioinformatics analysis of lncRNAs enriched in coronary thrombus-derived exosomes **(A)** Heat map showing the expression of DElncRNAs in TEs compared with those in healthy human serum exosomes. Each row in the heat map represents a single gene, and each column represents a tissue sample. The colour scale, ranging from blue (low expression) to red (high expression), represents the raw Z-score mRNA intensity values; **(B)** Volcano plot of DElncRNAs between TEs and healthy human serum exosomes. The red nodes represent up-regulated DEGs and the green nodes represent down-regulated DEGs; **(C)** Heat map showing the expression of DEmRNAs in TEs compared with those in healthy human serum exosomes; **(D)** Volcano plot of DEmRNAs between TEs and healthy human serum exosomes; **(E, F)** Circle chart and bubble diagram of gene ontology (GO) analysis of DEmRNAs showing enriched biological processes, cell components, and molecular functions; **(G, H)** Circle chart and bar chart of Kyoto Encyclopedia of Genes and Genomes (KEGG) pathway analysis of targeted genes showing enriched signaling pathways; **(I)** Schematic diagram of the pathways involved in ferroptosis-related genes based on KEGG analysis; **(J)** RT-PCR analysis of DElncRNA expression in each group of exosomes(n=3); **(K)** Predicted binding of FENDRR and P53 in each cell from the AnnoLnc database. a, compared with the HE group, *p* < 0.05; b, compared with the SE group, *p* < 0.05 HE, serum exosomes from healthy individuals; SE, serum exosomes from patients with AMI; TE, coronary thrombus-derived exosomes from patients with AMI.

To clarify the effector substances in TEs, we sent TEs for lncRNA-microarray analysis. Human SEs from healthy volunteers (n = 3) and coronary TEs from patients with AMI (n = 4) were analysed using lncRNA microarrays. Differential expression analysis using the Limma R package identified 2759 DElncRNAs, of which 2248 were up-regulated and 511 were down-regulated, and 1298 DEmRNAs, of which 1143 were up-regulated and 155 were down-regulated (**Figures 3A–D**). We have primarily explored the effects of TEs on cardiomyocytes and not of T1MI on thrombus-derived exosomes. Therefore, the bioinformatics analyses only investigated the highly expressed genes within the exosomes. We explored the relevant pathways associated with DElncRNAs and DEmRNAs by GO analysis, which indicated that the cellular component section revealed that these DEmRNAs(DIS3, EXOSC9, PNPT1, and DIS3L2) in TEs are involved in the regulation of the relevant pathways(**Figures 3E, F**). Subsequent KEGG analysis identified only four differential pathways, namely herpes simplex virus 1 infection, retinol metabolism, ferroptosis, and phototransduction (in descending order according to the number of DEmRNAs involved) (**Figures 3G–I**). Among these, only ferroptosis is considered to play a role in cardiovascular disease. KEGG analysis identified five DEmRNAs involved in ferroptosis, including solute carrier family 3 member A2 (SLC3A2), ceruloplasmin (CP), MAP1LC3C, ferritin heavy chain 1 (FHT1), and glutathione cysteine ligase modulatory subunit (GCLM).

To identify the most influential DElncRNA, we divided the subsequent samples into three groups, namely healthy human serum exosomes (HE group), serum exosomes from patients with AMI (SE group), and thrombus-derived exosomes from patients with T1MI (TE group). RT-PCR was used to analyse the top 20 upregulated RNAs, including five coding genes and 15 lncRNAs. In the TE group, ten lncRNAs exhibited upregulated expression relative to the HE group (*p*<0.05) (**Figure 3J**). Among them, LOC101928697, AC079341.1, and FENDRR expression were the highest, with quantification cycle (Cq) values of approximately 20. As these three lncRNAs were enriched within TEs, we on several lncRNA prediction websites(http://annolnc.gao-lab.org/and https://ngdc.cncb.ac.cn/lncexpdb/) and found that only FENDRR may be involved in ferroptosis. According to the AnnoLnc database (http://annolnc.gao-lab.org/index.php) predictions, FENDRR may bind directly to TP53 in numerous cell lines (**Figure 3K**), but the specific mechanisms by which FENDRR and P53 are functioning remain unclear. For this reason, we performed follow-up experiments to verify the effects of FENDRR and P53 in TE for cardiomyocytes.

### 3.4 TEs promote expression of lncRNAs FENDRR and P53 in cardiomyocytes

**Figure 4:**
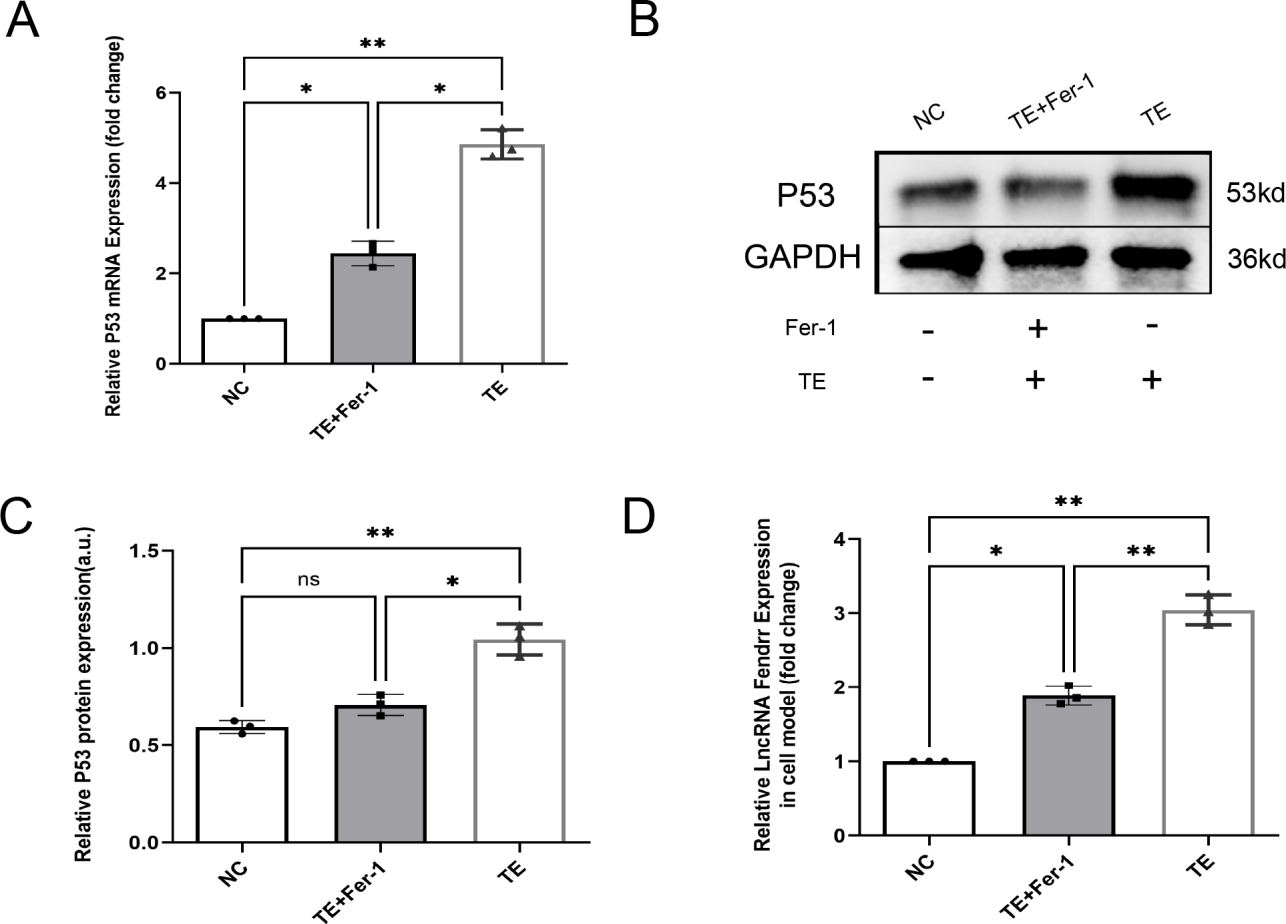
TEs promote the expression of lncRNAs FENDRR and P53 in cardiomyocytes(n=3). **(A)** RT-PCR detection of P53 mRNA expression in cardiomyocytes after TE treatment; **(B, C)** RT-PCR detection of P53 protein expression in cardiomyocytes after TE treatment; **(D)** RT-PCR detection of lncRNA FENDRR expression in cardiomyocytes after TE treatment. *, *p* < 0.05; **, *p* < 0.01. NC group, negative control group; TE group: myocardial cells co-cultured with coronary thrombus-derived exosomes from patients with AMI at 128 ng/µL for 24 h; TE+Fer-1 group, Fer-1 pre-treated myocardial cells co-cultured with TEs.

Bioinformatics analyses were used to screen lncRNAs and potential effector proteins and genes. In addition to high lncRNA FENDRR expression in TEs, cardiomyocytes treated with TEs exhibited significantly elevated P53 protein and mRNA expression compared with the NC group (*p* < 0.05) (**Figures 4A–C**). Fer-1 mitigated these effects (*p* < 0.05). Additionally, lncRNA FENDRR expression was markedly higher in TE-treated cardiomyocytes (*p* < 0.05) (**Figure 4D**). These results support the hypothesis that TEs play a vital role in regulating ferritinophagy by carrying the lncRNA FENDRR in cardiomyocytes.

### 3.5 LncRNA FENDRR synergises with P53 to regulate ferritinophagy in cardiomyocytes

**Figure 5:**
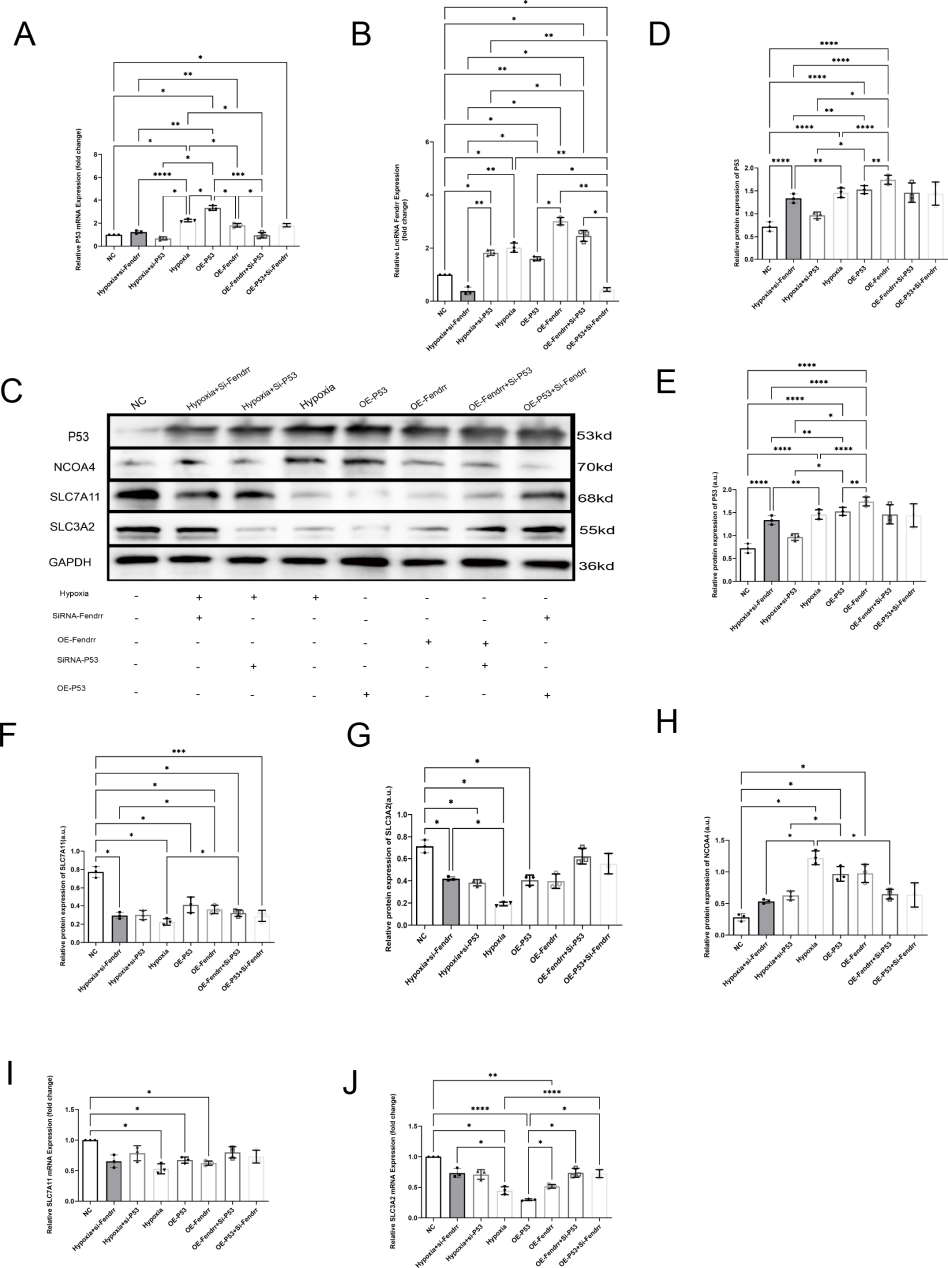
LncRNA FENDRR synergises with P53 to regulate ferritinophagy in cardiomyocytes(n=3). **(A)** RT-PCR results of P53 mRNA expression in the cell models,**(B)** RT-PCR results of lncRNA FENDRR;**(C)**Western blot of ferritinophagy-related protein expression; Western blot results of **(D)** P53, **(E)** NCOA4, **(F)** SLC7A11, and **(G)** SLC3A2 expression in the cell models; RT-PCR results of **(H)** NCOA4, **(I)** SLC7A11, **(J)** SLC3A2 mRNA expression in the cell models. *p < 0.05; **p < 0.01; ***p < 0.001; ****p < 0.0001. NC group, negative control group; Hypoxia group, positive control group with acute hypoxia induced by liquid paraffin for 2 h; OE-FENDRR group, model groups with lentiviral overexpression of lncRNA FENDRR; OE-P53 group, model groups with lentiviral overexpression of P53; Hypoxia+si-FENDRR group, inhibition of lncRNA FENDRR expression using siRNA and establishment of a cellular model of acute hypoxia; Hypoxia+si-P53 group, inhibition of P53 expression using siRNA and establishment of a cellular model of acute hypoxia; OE-P53+si-FENDRR group, inhibition of lncRNA FENDRR expression by siRNA in a cardiomyocyte model overexpressing P53; OE-FENDRR+si-P53 group, inhibition of P53 expression by siRNA in a cardiomyocyte model overexpressing the lncRNA FENDRR.

To verify that lncRNA FENDRR and P53 play important roles in cardiomyocyte ferritinophagy, we performed lncRNA FENDRR and p53 overexpression by the lentivirus and silence by the siRNA. We also established an acute ischemic-hypoxic cardiomyocyte model as a positive control group to simulate the *in vivo* microenvironment during AMI to gain a deeper understanding of the role of these genes in AMI (**Figures 5A–G**). At the RNA level (**Figures 5A, B**), there is no significant correlation between the expression of P53 and the lncRNA Fendrr (*p* > 0.05). At the protein level, we found that overexpression of either P53 or lncRNA FENDRR significantly promotes NCOA4 expression at both the protein level (**Figure 5E**) (*p* < 0.05) and inhibits SLC7A11 expression (*p* < 0.05) (**Figure 5F**). However, only the overexpression of P53 shows a significant effect on SLC3A2 (*p* < 0.05) (**Figure 5G**). NCOA4 (**Figure 5H**) and SLC37A11 (**Figure 5I**) mRNA expression were also consistent with the protein levels (*p* < 0.05). Upregulation of NCOA4 and downregulation of SLC3A2 (**Figures 5G, J**) and SLC7A11 (**Figures 5F, I**) were also seen in the acute ischemic-hypoxic cardiomyocyte model (*p* < 0.05). These results confirm that FENDRR can act synergistically with P53, which in turn regulates NCOA4 and system Xc expression. In combination with the predicted results from the NCBI database, we therefore speculated that direct binding may occur between FENDRR and P53.

### 3.6 LncRNA FENDRR synergises with P53 to regulate TEs-induced ferritinophagy effects in cardiomyocytes

**Figure 6:**
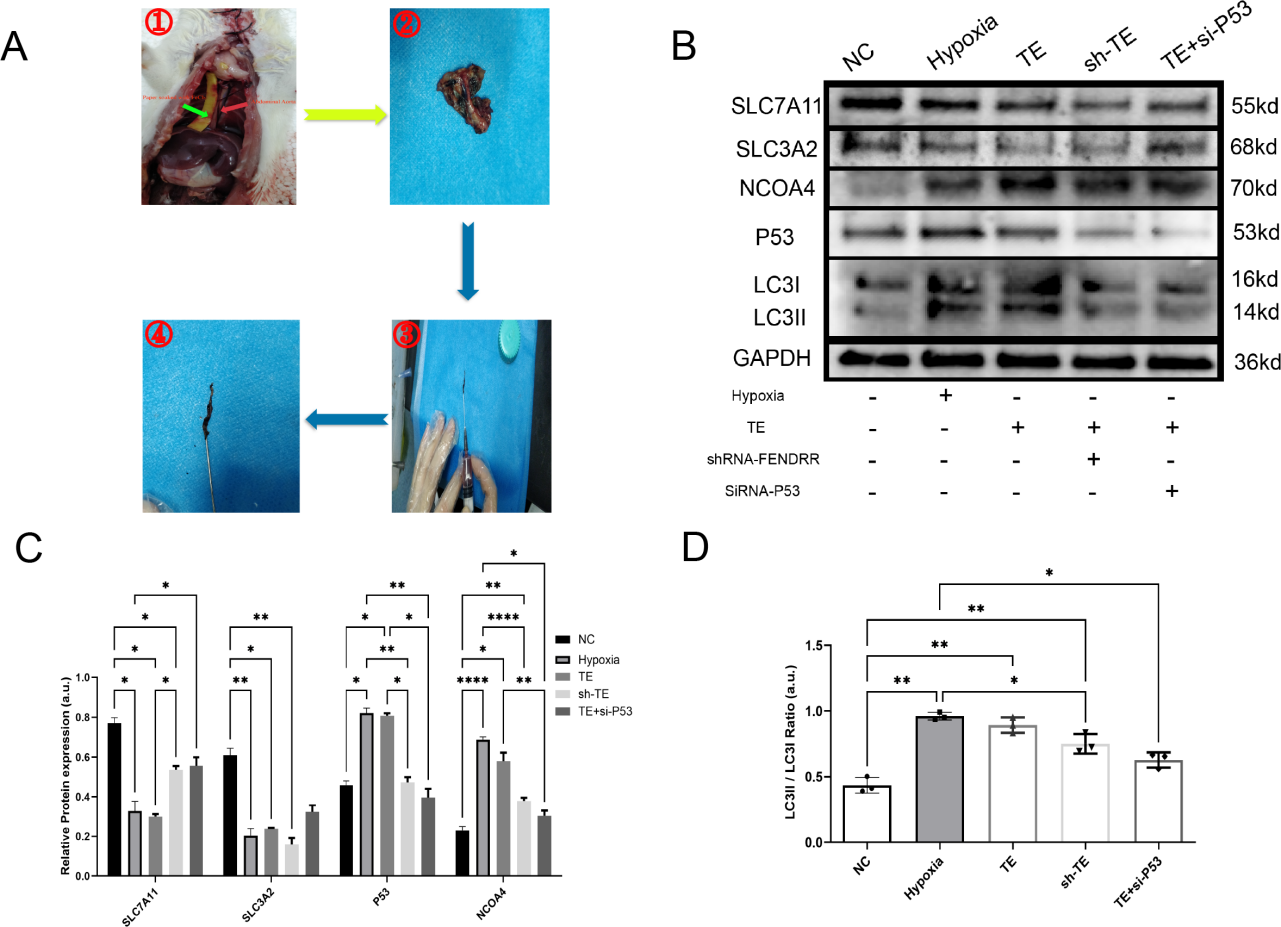
LncRNA FENDRR synergises with P53 to regulate TEs-induced ferritinophagy effects in cardiomyocytes(n=3). **(A)** Thrombus production in rats showing the strip of paper infiltrated with FeCl_3_ (green arrow) and the rat thoracic aorta (red arrow). These images are only representative of the procedure, as the rat thoracic aorta is located behind the lungs. For this study, the experiment was conducted with live rats. After approximately 3 minutes of thrombus formation in the thoracic aorta, a needle was used to flush out the thrombus for exosome extraction; **(B)** Western blots of ferritinophagy-related proteins in TE-treated cardiomyocyte models. AAV9 was used to construct lncRNA FENDRR-free TEs (sh-TE), and siRNA was used to inhibit P53 expression in cardiomyocytes co-cultured with TEs; **(C)** Western blot results of ferritinophagy-related protein expression; **(D)** Histogram of the ratio of LC3II to LC3I. *p < 0.05; **p < 0.01; ****p < 0.0001. NC group, negative control group; Hypoxia group, positive control group with acute hypoxia induced by liquid paraffin for 2 h; TE group: myocardial cells co-cultured with coronary thrombus-derived exosomes from patients with AMI at 128 ng/µL for 24 h; sh-TE group, local injection of AAV9-shRNA FENDRR into rat tail vein, followed by establishment of a T1MI rat model using FeCl_3_ and extraction of lncRNA FENDRR-free TEs and subsequent co-culture of these TEs with H9c2 myocardial cells; TE+si-P53 group, inhibition of P53 expression using siRNA in myocardial cells co-cultured with TEs.

In the previous sections, we preliminarily demonstrated that TEs can induce ferritinophagy in cardiomyocytes and largely confirmed the important role of FENDRR and P53 in ferritinophagy. We then investigated the effect of TEs on ferritinophagy induced via FENDRR and P53. We locally injected adeno-associated virus-9 (AAV-9) carrying shRNA-FENDRR into the tail vein of rats to establish a rat model of lncRNA FENDRR inhibition(n=6). We established a rat model of acute thrombosis using FeCl_3_ and collected the thrombi to extract exosomes (**Figure 6A**). We successfully extracted lncRNA FENDRR-free TEs (**Figure 6B**). The Western blot results showed that there were no significant differences in the LC3II/I ratio (**Figures 6C, D**) between the sh-TE group and the TE+si-P53 group compared to the TE group (*p >*0.05). Notably, compared to the TE group, the expression level of P53 protein was significantly downregulated in the sh-TE group (*p* < 0.05), while the expression of SLC7A11 protein was significantly upregulated (*p* < 0.05) (**Figures 6C, E**). Correspondingly, the expression level of NCOA4 protein was significantly downregulated in the TE+si-P53 group compared to the TE group (*p* < 0.05). Our results, therefore, confirmed that FENDRR and P53 play pivotal roles in the regulation of cardiomyocyte ferritinophagy induced by TEs.

### 3.7 Validation of the mutual binding of FENDRR and P53

**Figure 7:**
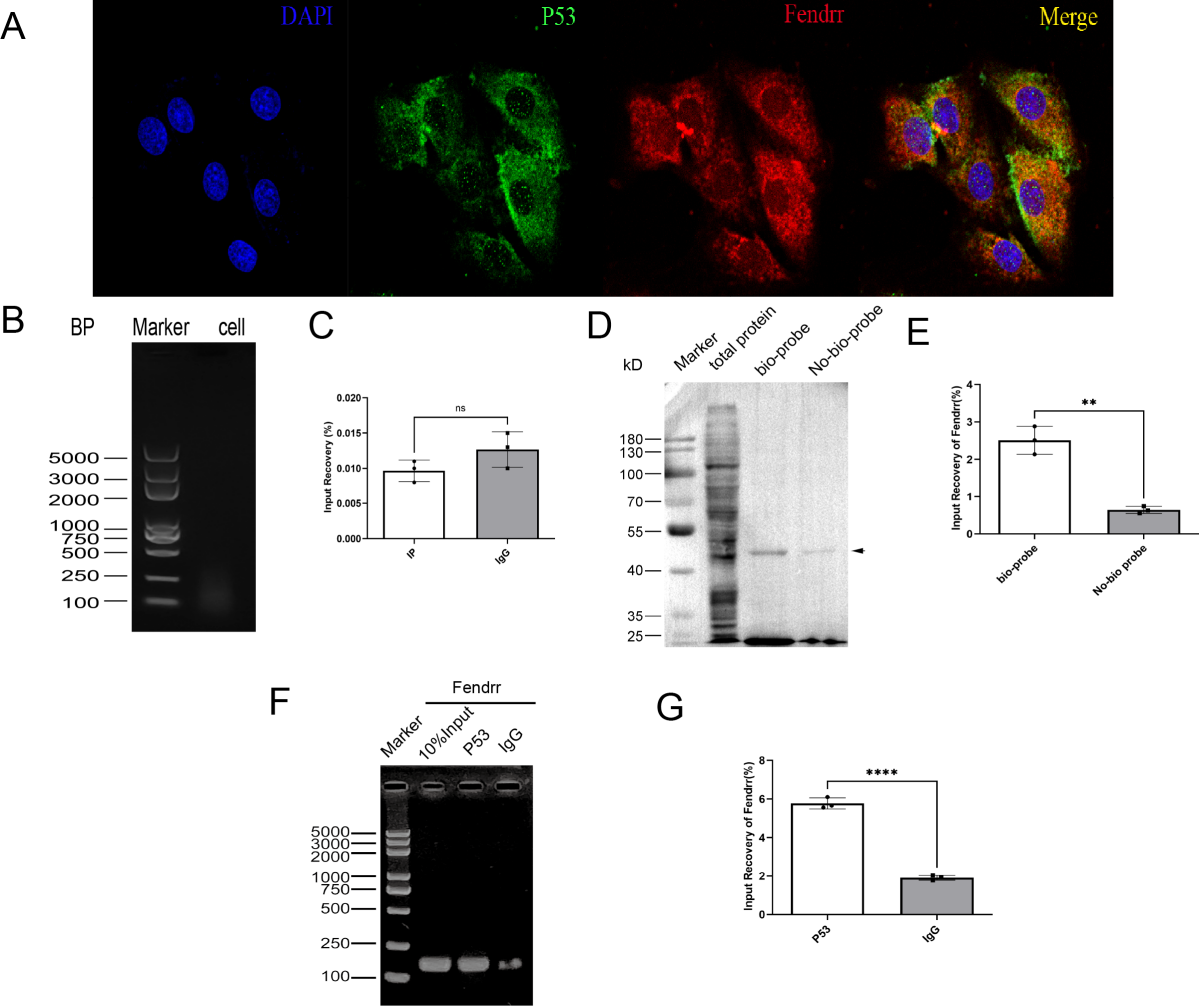
Validation of the mutual binding of FENDRR and P53(n=3). **(A)** FISH assay showing FENDRR and P53 promoter binding (60×); **(B, C)** ChIP assay showing the binding between FENDRR and the P53 promoter; **(D, E)** RNA-pulldown assay showing the binding between FENDRR and P53 protein; **(F, G)** RIP assay showing the binding between P53 protein and FENDRR. ns: not significant; **p < 0.01; ****p < 0.0001.

Although FENDRR was predicted to bind to P53 on the Annolnc website, its specific binding mode was not investigated. Fluorescent labelling of FENDRR (red) and P53 (green) in FISH experiments indicated partially overlapping FENDRR and P53 localisation (**Figure 7A**), which suggests that they may bind at the gene level. We subsequently identified the potential pooling site of the TP53 in the FENDRR promoter region as chr19:53010491-53010822. Based on the corresponding gene sequences, we designed the corresponding primers for ChIP experiments. CHIP assay demonstrated protein-DNA interaction, indicating that FENDRR does not bind to the p53 promoter region (*p* > 0.05) (**Figures 7B, C**). We then used the RNA pull-down technique to verify whether FENDRR binds directly to the P53 protein via RNA-binding protein (RBP) effects. P53 expression was significantly decreased in the no-bio-probe group compared with the bio-probe group (*p* < 0.05) (**Figures 7D, E**). Furthermore, we used the RIP technique to reverse-verify direct binding between FENDRR and the P53 protein and found that FENDRR expression is significantly decreased in the IgG group compared with that in the P53 group (*p* < 0.05) (**Figures 7F, G**). Our results, therefore, confirmed that FENDRR binds directly to P53 via RBP effects.

### 3.8 FENDRR and P53 complexes regulate m6A methylation modification in cardiomyocytes via YTHDF proteins

**Figure 8:**
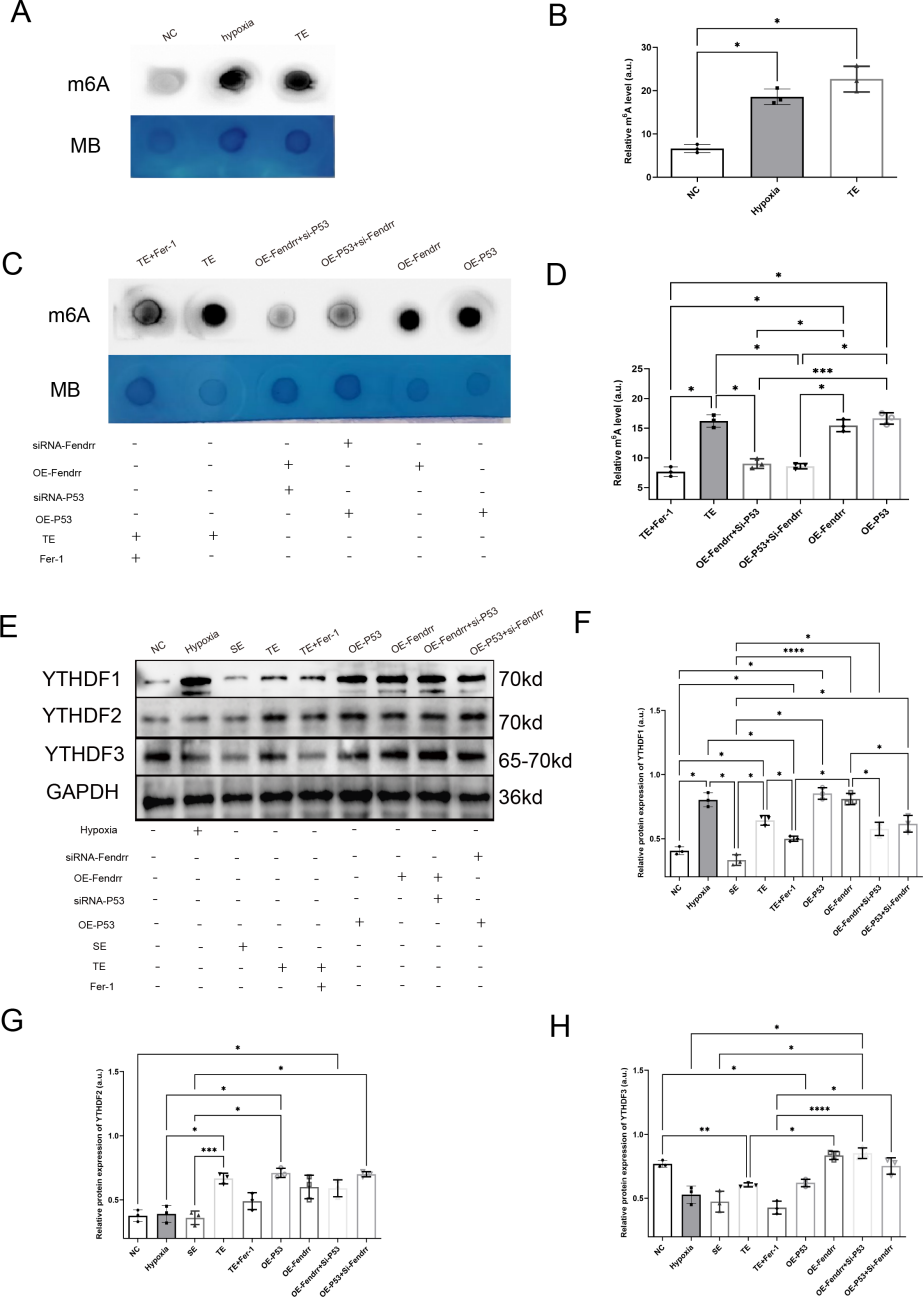
FENDRR and P53 complexes regulate m6A methylation modification in cardiomyocytes via YTHDF proteins(n=3). **(A)** m6A dot blot analysis of total RNA from cardiomyocytes co-cultured with TEs; **(B)** Dot blot analysis results of m6A methylation modification; **(C)** Dot blot analysis of total RNA samples from cardiomyocytes in both the lncRNA FENDRR and P53 overexpression/knockdown models; **(D)** Dot blot analysis results of m6A methylation modification; **(E)** Western blot of YTHDF protein expression in cardiomyocytes in lncRNA FENDRR and P53 overexpression/knockdown models; Western blot results of **(F)** YTHDF1, **(G)** YTHDF2, and **(H)** YTHDF3 protein expression. *p < 0.05; **p < 0.01; ***p < 0.001; ****p < 0.0001. MB, methylene blue (loading control); NC group, negative control group; Hypoxia group, positive control group with acute hypoxia induced by liquid paraffin for 2 h; OE-FENDRR group, model groups with lentiviral overexpression of lncRNA FENDRR; OE-P53 group, model groups with lentiviral overexpression of P53; OE-P53+si-FENDRR group, inhibition of lncRNA FENDRR expression by siRNA in a cardiomyocyte model overexpressing P53; OE-FENDRR+si-P53 group, inhibition of P53 expression by siRNA in a cardiomyocyte model overexpressing the lncRNA FENDRR; TE group, myocardial cells co-cultured with coronary thrombus-derived exosomes from patients with AMI at 128 ng/µL for 24 h; TE+Fer-1 group, Fer-1 pre-treated myocardial cells co-cultured with TEs; SE group, myocardial cells co-cultured with serum derived exosomes at 128 ng/µL for 24 h.

By referring to the RNA INTER database (rnainter.org), we found that FENDRR and P53 have the potential to bind to the YT521-B homology domain family (YTHDF) proteins. We therefore analysed the expression of m6A in cardiomyocytes and found significantly increased m6A expression in cardiomyocytes co-cultured with TEs (*p* < 0.05) (**Figures 8A, B**). Conversely, the incorporation of Fer-1 into TE markedly attenuated the elevation of m6A expression resulting from TE(*p* < 0.05) (**Figures 8C, D**). It is worth mentioning that the overexpression of either P53 or FENDRR significantly increases the expression of m6A in cardiomyocytes, and the expression level shows no significant difference compared to the TE group(*p* > 0.05). Similarly, when the expression of P53 or FENDRR is inhibited in the corresponding groups, there is a significant reduction in the expression of m6A in cardiomyocytes (*p* < 0.05) (Figurea 8C, D). We also investigated the expression of YTHDF1/2/3 proteins in a cardiomyocyte model (**Figures 8E–H**). The results showed that TE treatment significantly increased YTHDF1 expression (*p* < 0.05) while inhibiting YTHDF3 expression (*p* < 0.05). In contrast, treatment with Fer-1 notably improved the differential expression of YTHDF1 and YTHDF3 induced by TEs (*p* < 0.05). Our findings also highlight the importance of P53 in this process. Compared to the NC group, overexpression of P53 significantly upregulated YTHDF1 and downregulated YTHDF3 (*p* < 0.05), with a pattern similar to that observed in the TEs group. Our results therefore confirm that P53 act synergistically to regulate m6A methylation via the YTHDF family in cardiomyocytes.

### 3.9 Single-cell analysis of coronary arterial plaque tissue

**Figure 9:**
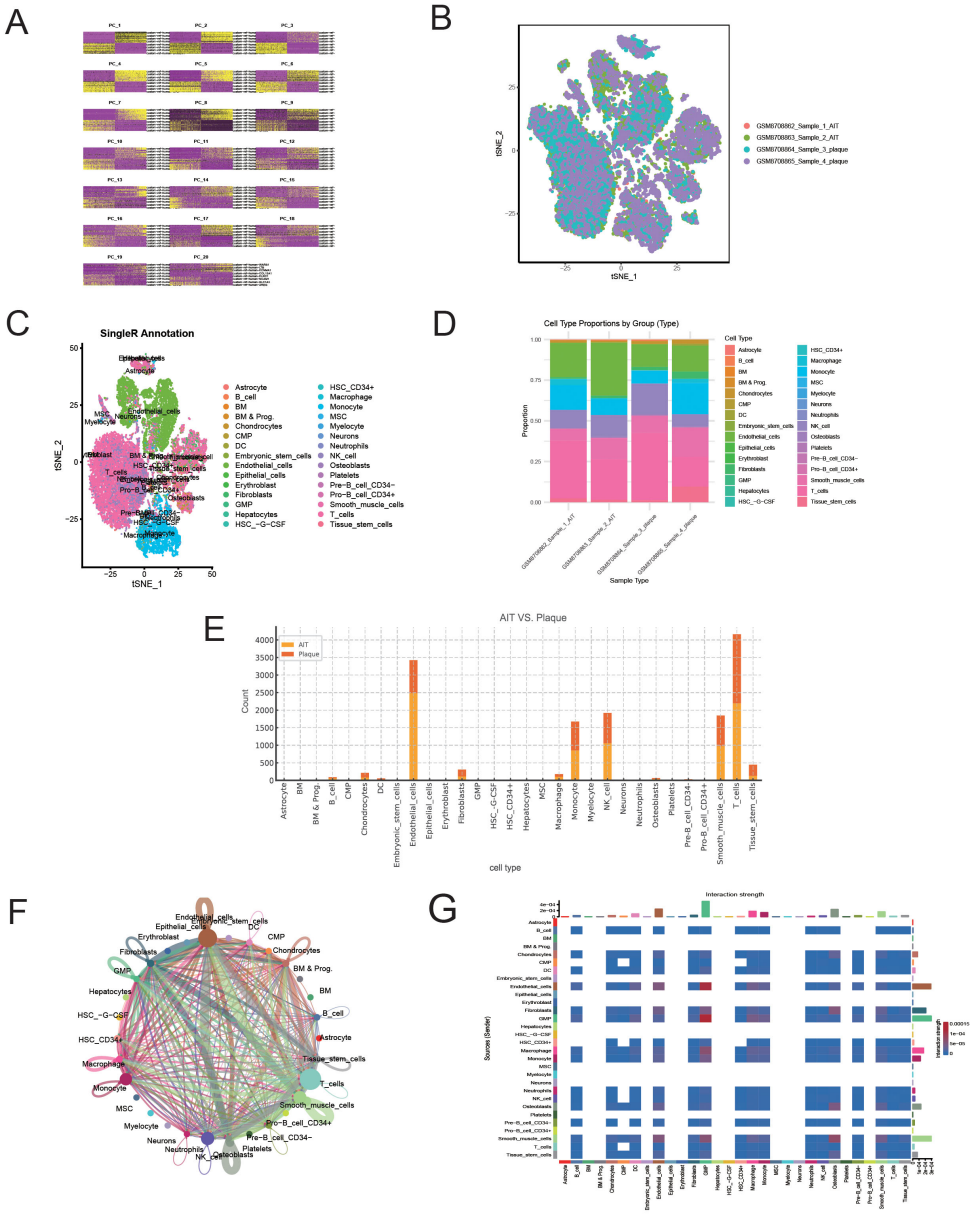
Single-cell RNA sequencing reveals cellular heterogeneity and interaction patterns between atherosclerotic plaques and aortic tissue. **(A)** Quality control metrics of the sequencing samples. **(B)** t-SNE plot visualising the distribution of cells in each sample. **(C)** Cell type annotation based on the SingleR algorithm. **(D)** Comparison of the cell composition ratios between different tissue sources. **(E)** Distribution of cell types between AT (aorta) and Plaque tissue. **(F)** Cell-cell communication network diagram (CellChat analysis). **(G)** Heatmap of cell-cell communication intensity.

To further explore the correlation between T1MI and ferritinophagy, we performed single-cell analysis using the publicly available AS-related dataset (GSE285775) (**Figures 9A, B**). In this single-cell data, Arterial non-plaque tissue (AIT group) was used as the control, and plaque tissue was the experimental group. As shown in **Figures 9C–E**, T cells were the most predominant cell population in AS plaque tissue, accounting for 26.95% of the total in the AIT group versus 31.03% in the plaque. Similarly, monocytes (10.49% vs. 12.94%) and NK cells (13.01% vs. 13.55%) also made up substantial proportions. These results suggest a high presence of inflammatory and immune cells in AS plaques, indicating significant local immune infiltration and inflammatory responses. Notably, although nearly all immune cells showed a marked increase in plaque tissue, the proportion of macrophages decreased significantly (1.30% vs. 1.13%). To further clarify this, we conducted additional analyses. We also performed intercellular interaction network analysis based on the differentially expressed genes (DEGs) of each cell component. Our findings revealed that macrophages exhibited high connectivity within the interaction network, forming dense connections with neutrophils, monocytes, T cells, and other immune cells, indicating that macrophages play a central role in signal transmission within the inflammatory microenvironment (**Figure 9F**). The interaction intensity of macrophages was significantly higher than that of other immune cell types, such as neutrophils or T cells, with both signal sending and receiving abilities at their peak, suggesting that macrophages drive both the activation and resolution of inflammation (**Figure 9G**).

### 3.10 Single-cell analysis of macrophage DEGs reveals enrichment of ferroptosis- and autophagy-related pathways in T1MI

**Figure 10:**
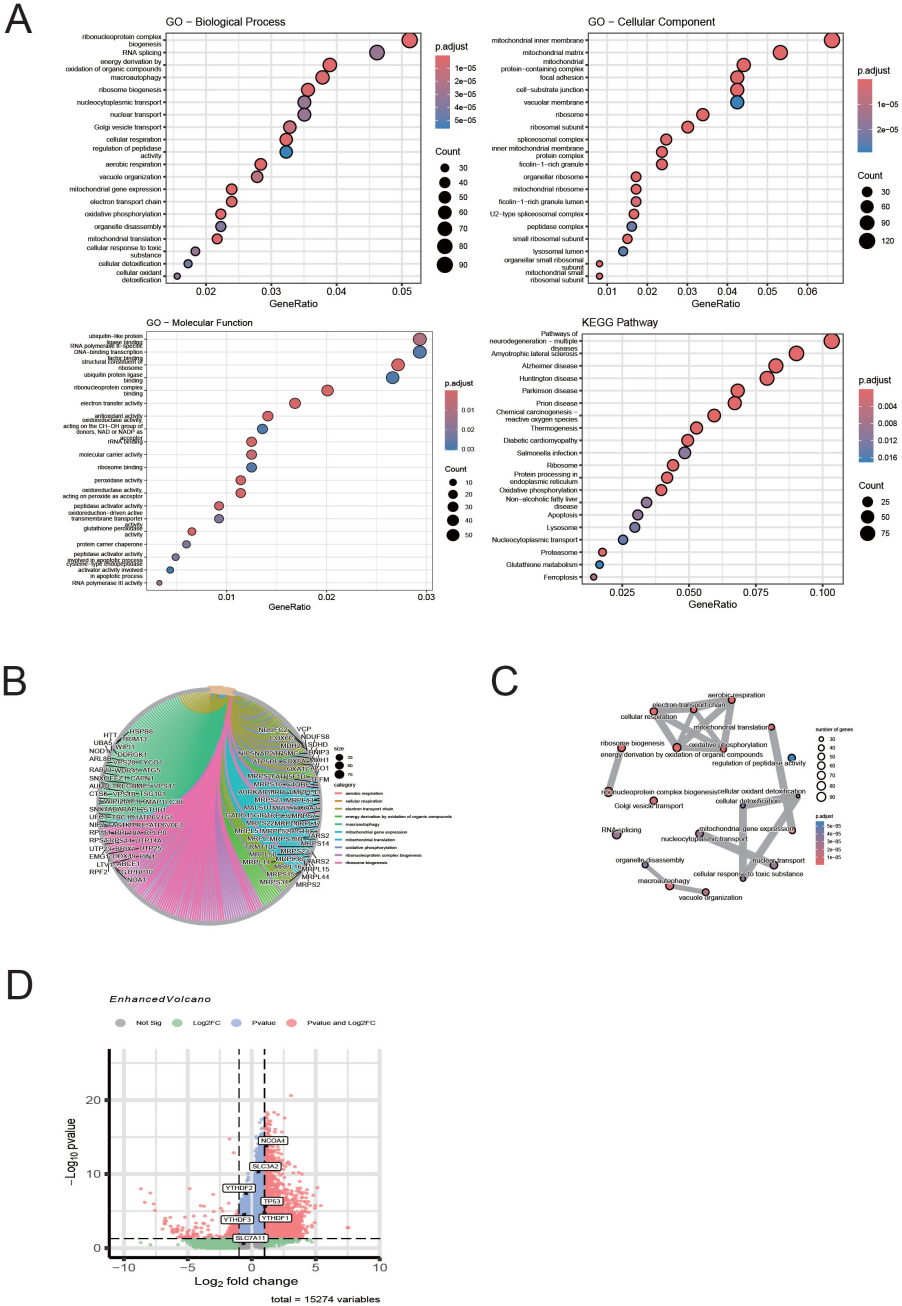
Single-cell analysis of macrophage DEGs reveals enrichment of ferroptosis- and autophagy-related pathways in T1MI. **(A)** GO and KEGG enrichment analyses show that the differentially expressed genes (DEGs) in macrophages are mainly enriched in pathways related to mitochondrial metabolism, protein translation, autophagy, and ferroptosis. **(B)** The chord diagram illustrates the correspondence between DEGs and GO terms. **(C)** The GO network graph reveals the association structure between functional terms, suggesting the synergistic effect of multiple biological pathways. **(D)** The volcano plot highlights the ferritinophagy genes (NCOA4, SLC3A2, SLC7A11, TP53) and m6A-related genes (YTHDF1/2/3) of interest in this study, with NCOA4, TP53, and YTHDF1 showing significant upregulation in macrophages.

To further explore the relationship between macrophage-associated immune-inflammatory responses, ferroptosis, and autophagy, we conducted functional annotation and pathway enrichment analysis of differentially expressed genes (DEGs) in macrophages based on single-cell data, following our previous confirmation of ferritinophagy in T1MI (**Figure 10**). In the GO analysis (**Figure 10A**), significant enrichment was observed in biological processes (BP) related to metabolic and energy conversion pathways, including mitochondrial oxidative metabolism, oxidative phosphorylation, and ribosome biogenesis. In terms of molecular functions (MF), the enrichment was focused on rRNA binding and structural molecular activity. For cellular components (CC), the enrichment was found in organelles such as the mitochondrial inner membrane, ribosomes, and lysosomes. These results suggest that in T1MI, macrophage function is heavily reliant on metabolic reprogramming and dynamic changes in organelles. KEGG pathway analysis (**Figure 10A**) showed significant enrichment in pathways closely associated with ferritinophagy, including “ferroptosis,” “autophagy,” and “glutathione metabolism,” further supporting the core role of ferritinophagy in this process. **Figures 10B, C** present the relationship network between DEGs and enriched GO pathways (Chord Diagram), clearly showing that multiple key DEGs are involved in various important biological processes and functional modules, highlighting their multifaceted roles in regulating immune metabolism and ferritinophagy. Notably, in the volcano plot in **Figure 10D**, we specifically highlighted key genes closely related to ferritinophagy, including NCOA4, SLC3A2, SLC7A11, and TP53, as well as m6A-related regulatory proteins YTHDF1/2/3. Among these, NCOA4, TP53, and YTHDF1 were significantly upregulated in macrophage DEGs, suggesting that these genes may activate the ferritinophagy pathway in T1MI-associated macrophages, accompanied by m6A modification regulatory mechanisms at the epigenetic level. This finding aligns with our previous results obtained from exosome chip data, further emphasising the core regulatory role of NCOA4 and TP53 in the cross-talk between ferroptosis and autophagy.

In summary, through single-cell immune cell fine typing and functional pathway analysis, this section reveals the potential mechanism by which macrophages in T1MI link immune response, metabolic regulation, and cell death via ferritinophagy, highlighting the significance of the TP53–NCOA4 axis and m6A regulatory proteins in this pathological process.

## 4 Discussion

To our knowledge, this is the first study to elucidate the regulatory role of thrombus-derived exosomes (TEs) in ferritinophagy within cardiomyocytes. Building upon our previous work (36), we further demonstrate that TEs can significantly modulate P53 expression in cardiomyocytes, primarily through the delivery and enrichment of the lncRNA FENDRR. This newly identified pathway coordinates several crucial cellular processes, including ferroptosis-associated autophagy, apoptosis, inflammation, and immune activation, thereby highlighting a novel mechanistic link between exosomal signaling and myocardial injury.

Ferritinophagy, an autophagy-dependent type of ferroptosis, has emerged as a critical area of cardiovascular research in recent years (37). Disruptions in the balance between intracellular lipid oxidation and ROS metabolism lead to ferroptosis, which often coincides with increased autophagic activity (38, 39). Overload of intracellular iron can trigger ferroptosis through two main pathways: free Fe²^+^ catalyzes the Fenton reaction, producing hydroxyl radicals and driving oxidative stress; at the same time, Fe²^+^ acts as a cofactor for metabolic enzymes that amplify lipid ROS production and further promote cell death (40, 41). Our findings are in line with these mechanisms. In our experiments, TEs-induced ferritinophagy in cardiomyocytes was characterized by elevated autophagy, increased ROS, and enhanced lipid peroxidation.The concept of ferritinophagy was introduced by Mancias et al. (42) in 2014, first identified NCOA4 enrichment in autophagosomes. Since then, NCOA4, together with endogenous LC3B, intracellular iron stores, and ferritin complexes, has been linked to this process. Dysregulated ferritinophagy, primarily driven by excess ferrous ions (43) and leads to harmful lipid oxidation (44) and ROS damage (45). These reactions have been implicated in conditions such as cardiomyocyte hypertrophy (46), chronic obstructive pulmonary disease (47), and multiple myeloma (48). Consequently, ferritinophagy and its influence on physiological and disease processes have received considerable attention. Autophagy is known to be essential for cardiac function and homeostasis, particularly in post-mitotic cells such as adult cardiomyocytes (49). Under stress, protein aggregates are cleared by autophagic mechanisms involving ubiquitination of SQSTM1/p62 and LC3 activation, both of which play important roles in cardiac diseases, including AMI (50), atherosclerosis (51), and HF (52). Ferroptosis is a complex mechanism that induces cell death by catalysing the peroxidation of highly expressed unsaturated fatty acids on cell membranes in the presence of divalent iron or ester oxygenases (53). In addition, ferroptosis is manifested by a decrease in the activity of the regulatory core enzyme GPX4 of the glutathione antioxidant system (54). System Xc-, comprising SLC3A2 and SLC7A11, is central to maintaining intracellular iron homeostasis (55). P53 regulates iron-dependent cell death through mechanisms independent of GPX4 and system Xc- (56). In our study, TEs increased Fe²^+^ and MDA, decreased SOD, upregulated NCOA4, and inhibited system Xc-. These findings, together with observed alterations in autophagy and apoptosis, strongly indicate that TEs promote cardiomyocyte ferritinophagy.

The relationship between AMI and ferritinophagy is not fully understood. Some studies have suggested that cyanidin-3-glucoside (C3G) protects cardiomyocytes in AMI by inhibiting NCOA4 and LC3II/I expression (57), suggesting a role for ferritinophagy in this process. Recent bioinformatic analyses have identified a set of ferroptosis- and autophagy-related genes, including Prostaglandin-Endoperoxide Synthase 2(PTGS2), Jun Proto-Oncogene, AP-1 Transcription Factor Subunit(JUN), NAD(P)H Quinone Dehydrogenase 1(NQO1), Nitric Oxide Synthase 3(NOS3), and Leptin Receptor(LEPR), as potential therapeutic targets in myocardial infarction and for reducing adverse cardiovascular events (58). While Fer-1 was used in our experiments as a classical inhibitor of ferritinophagy/ferroptosis and showed cytoprotective effects, it is important to note that as a lipid peroxidation inhibitor, Fer-1 can also affect multiple signaling pathways, including those related to oxidative stress and cell death. Thus, the protective effects seen with Fer-1 in this context should not be considered entirely specific for ferritinophagy inhibition, and these results should be interpreted cautiously. Future studies should incorporate more targeted genetic interventions alongside highly specific inhibitors to clarify the independent role of ferritinophagy in myocardial injury.

The significance of lncRNAs, especially those delivered by exosomes, has become increasingly apparent in cardiovascular biology. For example, lncRNA 2810403D21Rik/Mirf is involved in cardiomyocyte autophagy during AMI (59), and P53-related RNA-binding protein-associated lncRNA (P53RRA) regulates ferroptosis in tumor cells by directly binding to P53 (60, 61). Among the differentially expressed lncRNAs identified in our screen of TEs, FENDRR (also known as FOXF1-AS1) stood out due to its established roles in both development and disease. As comprehensively described by Szafranski et al. (62), FENDRR is a structurally complex, highly conserved long non-coding RNA located at 16q24.1, giving rise to multiple alternatively spliced transcripts. Functionally, FENDRR acts as a molecular scaffold for chromatin-modifying complexes, regulates microRNA activity as a ceRNA, and influences the stability and translation of various mRNAs. Experimental evidence supports its critical involvement in embryonic heart and lung development, and its dysregulation has been associated with a range of pathologies, including various cancers, myocardial fibrosis (63), hypertension, and HF (64). Accumulating data also indicate that FENDRR may serve as a biomarker for cancer diagnosis, prognosis, and therapeutic response (65). In the context of our study, we observed that FENDRR interacts with P53 through RNA-binding proteins, thereby modulating the expression of NCOA4 and system Xc- to induce ferritinophagy in cardiomyocytes. Our data suggest that NCOA4 upregulation may follow suppression of system Xc-, offering further mechanistic insight into how FENDRR integrates exosomal signals with the regulation of iron homeostasis and cell death in the injured heart.

Post-transcriptional m6A modifications have increasingly been recognized as important contributors to cardiovascular disease pathophysiology, including cell proliferation, autophagy, apoptosis, and inflammation (66, 67). Jin et al. (68) found that lncRNA CACNA1G-AS1 affects the m6A modification of Ferritin Heavy Chain 1(FTH1) via regulation of Insulin Like Growth Factor 2 mRNA Binding Protein 1(IGF2BP1), which in turn affects ferritinophagy in ovarian cancer cells. The YTHDF family proteins, the best-known family of m6A RNA binding proteins, have an aromatic pocket structure that recognises and binds to m6A methylation sites. Within the YTHDF family, YTHDF1 increases ribosome occupancy and translation efficiency by directly interacting with translation initiation factors, while YTHDF2 promotes degradation of m6A-modified mRNAs (69). Several recent studies have confirmed that YTHDF2 can inhibit the expression of SLC7A11 and SLC3A2 through m6A modification, which in turn regulates ferroptosis in rectal cancer cells (70). Our study also confirmed that FENDRR lncRNA acts synergistically with P53, possibly by regulating the expression of YTHDFs, which in turn affects m6A methylation modification in cardiomyocytes and suppresses system Xc- expression.

In this study, we further validated the abnormal expression of key ferritinophagy factors (such as NCOA4 and TP53) and the m6A regulatory factor YTHDF1 in macrophages from T1MI patients through single-cell analysis, suggesting that immune cells play a crucial role in iron homeostasis regulation. Previous studies have shown that NCOA4-mediated ferritinophagy can enhance ferroptosis sensitivity by releasing free iron (21), while TP53 (71), as an upstream transcription factor, regulates multiple iron metabolism and autophagy-related genes (72). In the context of ferritinophagy, TP53 occupies a pivotal regulatory position owing to its dual role in iron-dependent cell death. On the one hand, TP53 can transcriptionally upregulate NCOA4, thereby promoting ferritin degradation, increasing the labile iron pool, and sensitising cells to ferroptosis (71). On the other, it can repress SLC7A11, a key component of system Xc⁻, leading to reduced glutathione synthesis and diminished antioxidant capacity, which further enhances lipid peroxidation (56). Notably, under certain stress conditions, TP53 may instead activate cell cycle arrest and DNA repair pathways, thereby mitigating excessive ferroptotic damage (54). In our study, thrombus-derived exosomes induced both NCOA4 upregulation and SLC7A11 downregulation in cardiomyocytes and macrophages, suggesting that, within the T1MI-associated immune microenvironment, TP53 predominantly exerts a pro-ferroptotic influence. This observation is consistent with prior evidence from oncology and cardiovascular research (72–74), where TP53 has been shown to toggle between cytoprotective and cytotoxic functions depending on upstream signalling cues, m6A-mediated RNA regulation, and the surrounding inflammatory microenvironment.

Importantly, the upregulation of YTHDF1 provides a new perspective on RNA modification-mediated ferritinophagy regulation. As a classic m6A reader protein, YTHDF1 enhances the translation efficiency of specific transcripts (75), and previous studies have reported its involvement in regulating the expression of genes related to ferroptosis (76). In our study, we found that YTHDF1 is co-upregulated with TP53 and NCOA4 in macrophages, suggesting that it may accelerate the initiation of ferritinophagy by promoting the translation of TP53 or NCOA4 transcripts. This mechanism links RNA epigenetic modifications, iron metabolism, and immune functions, offering a more comprehensive framework for understanding the pathogenesis of T1MI. It also provides potential directions for future interventions targeting RNA modification factors in immune-inflammatory responses.

While our study demonstrates the functional significance of thrombus-derived exosomes, it is important to acknowledge the limitations inherent to the experimental design, particularly the use of the FeCl_3_-induced thrombosis model and the lack of healthy or non-thrombotic controls. Addressing these issues in future research will be essential for a more comprehensive understanding and clinical translation of these findings.

## 5 Limitation

Despite the use of multi-omics data and single-cell analysis to systematically reveal the potential role of ferritinophagy in T1MI, there are still certain limitations in this study. Firstly, the sample size of TEs in this study is relatively small, which may lead to potential false positives. Secondly, m6A modification is a dynamic and highly complex epigenetic mechanism with intricate upstream and downstream regulation. Its specific regulatory role in different cell types remains unclear and warrants further investigation. Additionally, the single-cell analysis primarily focused on macrophages and did not cover the ferritinophagy characteristics of other immune cell subpopulations, which may have led to an underestimation of the mechanism’s role in the overall immune microenvironment. Moreover, as noted by the reviewer, the use of the FeCl_3_-induced rat model primarily for thrombosis induction, rather than for direct *in vivo* functional validation, and the lack of a healthy or non-thrombotic control group, may further limit the generalisability of our findings.

## 6 Conclusions

This study is the first to systematically reveal the potential core role of ferritinophagy in the pathogenesis of T1MI. By integrating exosome chip data with single-cell transcriptomic analysis, we identified the TP53–NCOA4 axis and the m6A regulatory factor YTHDF1 and YTHDF3 as key pathways that co-regulate ferroptosis and autophagy in immune cells, particularly macrophages. Our results suggest that ferritinophagy not only participates in the stress response within myocardial cells but may also mediate the initiation and amplification of inflammatory responses in immune cells. This study provides a new perspective on understanding iron homeostasis imbalance and immune pathological mechanisms in T1MI, laying a theoretical foundation for the future development of diagnostic and therapeutic strategies targeting ferritinophagy.

## Data Availability

The datasets presented in this study can be found in online repositories. The names of the repository/repositories and accession number(s) can be found below: https://www.ncbi.nlm.nih.gov/, GSE213115 https://www.ncbi.nlm.nih.gov/, GSE285775.
